# Predictive clinical indicators of refractory *Mycoplasma pneumoniae* pneumonia in children: A retrospective cohort study

**DOI:** 10.1097/MD.0000000000039375

**Published:** 2024-08-23

**Authors:** Hong Pei, Hongli Luo

**Affiliations:** a Department of Pharmacy, Hejiang People’s Hospital, Luzhou City, Sichuan Province, China; b Department of Clinical Pharmacy, The Affiliated Hospital, Southwest Medical University, Luzhou City, Sichuan Province, China.

**Keywords:** diagnostic indicators, pediatric pneumonia, predictive model, refractory *Mycoplasma pneumoniae* pneumonia, retrospective cohort study, ROC analysis

## Abstract

To determine the clinical indicators predictive of refractory *Mycoplasma pneumoniae* pneumonia (RMPP) in children and develop a robust predictive model to aid in early identification and management. A retrospective cohort study was conducted on 338 children diagnosed with RMPP out of a total of 1500 cases of *Mycoplasma pneumoniae* at a single tertiary hospital from May 2021 to November 2023. Clinical and demographic data analyzed included age, gender, parents’ educational level, household income, body mass index, allergic constitution, and laboratory findings such as white blood cell count, neutrophil and lymphocyte counts, platelet count, and levels of C-reactive protein (CRP), D-dimer, and procalcitonin. Univariate and multivariate logistic regression analyses were performed to identify significant predictors of RMPP, and a predictive model was developed. Among the RMPP cohort, 52.4% were female, with a mean age of 6.07 ± 2.78 years. Multivariate analysis identified several significant predictors of poor prognosis, including higher body mass index, longer duration of fever, elevated white blood cell count, neutrophil count, C-reactive protein levels, and increased neutrophil to lymphocyte ratio and platelet to lymphocyte ratio. The model demonstrated outstanding diagnostic performance, with an area under the receiver operating characteristic curve of 0.963 (95% confidence interval: 0.946–0.981). Our study identifies key clinical indicators with significant diagnostic accuracy for predicting RMPP in children. The predictive model established offers a valuable tool for clinicians, potentially improving RMPP outcomes through timely intervention.

## 1. Introduction

*Mycoplasma pneumoniae*, a common cause of community-acquired pneumonia in children, typically manifests with mild symptoms amenable to outpatient treatment.^[1–3]^ However, a subset of cases evolve into refractory *Mycoplasma pneumoniae* pneumonia (RMPP), characterized by persistent fever and radiographic abnormalities despite appropriate antibiotic therapy.^[4–6]^ The incidence of RMPP has risen globally, presenting a challenge to pediatric care.^[7–9]^

The pathophysiology of RMPP is complex, involving factors such as excessive immune response, macrolide resistance, and *Mycoplasma pneumoniae* virulence.^[10–12]^ This complexity is compounded by the variability in clinical presentation, ranging from mild respiratory symptoms to severe pneumonia, necessitating hospitalization, and aggressive treatment.^[13–15]^ Despite advances in diagnostic techniques, RMPP remains difficult to predict and manage effectively.^[16]^

Early identification of children at risk for RMPP is critical. Several studies have highlighted the potential role of demographic factors, clinical features, and laboratory markers as prognostic indicators.^[17,18]^ For instance, age and gender have been inconsistently associated with RMPP outcomes.^[19]^ In contrast, laboratory markers such as elevated white blood cell count, neutrophil predominance, and increased inflammatory markers like C-reactive protein (CRP) have shown a more consistent correlation with the disease severity.^[20]^

The development of a reliable predictive model for RMPP could improve the clinical approach to managing *M pneumoniae* infections. Predictive modeling may enable healthcare providers to identify children at high risk for poor outcomes and tailor treatment strategies accordingly.^[10]^ Such a model could integrate various prognostic indicators, potentially improving the specificity and sensitivity of RMPP predictions.

This study aims to bridge the existing knowledge gap by analyzing a comprehensive set of variables to construct a predictive model with the potential to forecast RMPP with high accuracy. By determining the clinical utility of various indicators and constructing a robust model, we hope to contribute to better patient stratification and individualized patient care in pediatric RMPP.

## 2. Materials and methods

### 2.1. Study design and population

This study analyzed a subset of 338 children diagnosed with RMPP from a larger cohort of 1500 patients treated for *M pneumoniae* pneumonia at a single tertiary hospital between May 2021 and November 2023. The inclusion criteria were as follows: (1) all patients met the diagnostic standards for *M pneumoniae* pneumonia as outlined in the eighth edition of “Zhufutang Practical Pediatrics”; (2) symptoms did not improve after more than 1 week of treatment; (3) pulmonary imaging indicated extensive consolidation areas with necrosis, and bronchoscopic examination revealed mucous plugs and mucosal necrosis. Exclusion criteria included: (1) severe tracheal/pulmonary maldevelopment or malformation; (2) congenital heart disease; (3) genetic metabolic diseases; (4) immunodeficiency disorders; (5) incomplete medical records.

### 2.2. Data collection and variables

Clinical data for this study were systematically extracted from the hospital’s electronic health records. The collected information included demographic details (age, gender), which are fundamental for assessing any age and gender-related variations in treatment response. We also included socioeconomic factors such as parental education levels and household income brackets to explore potential correlations with health outcomes and healthcare accessibility. The clinical data encompassed a range of variables critical for diagnosing and monitoring the progression of *M pneumoniae* pneumonia. These included full blood counts and differential counts (white blood cell [WBC] count, neutrophil and lymphocyte counts, platelet count) to evaluate the immune response to infection. Key derived ratios such as the neutrophil to lymphocyte ratio (NLR) and platelet to lymphocyte ratio (PLR) were calculated to provide insights into the systemic inflammatory response, which can be significant in severe infections like RMPP. Additionally, specific biomarkers were selected based on their proven diagnostic and prognostic relevance in inflammatory and infectious processes. CRP was included as a marker of inflammation, D-Dimer (DD) for assessing coagulation abnormalities often seen in severe infections, procalcitonin (PCT) as a marker more specific to bacterial infections, and lactate dehydrogenase (LDH) to gauge cellular damage and turnover.

Each variable was chosen not only for its clinical relevance but also for its potential to provide a comprehensive understanding of the factors that influence the severity and outcome of RMPP. This selection aims to enable a robust analysis of these variables’ impact on the prognosis of RMPP in children.

### 2.3. Outcome assessment

The primary outcome of this study was the prognosis of pneumonia, adjudicated as “good” or “poor” based on patient response to treatment. Prognosis was classified as “good” if patients demonstrated clinical recovery within 10 days of initiating appropriate antibiotic therapy. Recovery was defined by resolution of symptoms, improvement in radiological findings, and normalization of inflammatory markers. Conversely, prognosis was classified as “poor” if symptoms persisted or worsened, requiring additional therapeutic interventions beyond the initial treatment period. To ensure a consistent and unbiased evaluation, the assessment of each patient’s response was conducted by a panel of experienced pediatric pulmonologists. This panel reviewed comprehensive clinical data, including medical records, laboratory results, and imaging studies. The panel convened regularly to discuss and reach a consensus on each case, thereby standardizing the adjudication process across the cohort.

### 2.4. Ethical review

This retrospective cohort study was conducted in compliance with the ethical standards of the institutional and national research committee and with the 1964 Helsinki declaration and its later amendments or comparable ethical standards. Ethical approval for this study was granted by the Ethics Committee of Hejiang People’s Hospital, with the approval number 2023-003. Due to the retrospective nature of this analysis and the use of anonymized patient data, the requirement for informed consent was waived by the Ethics Committee. This decision was based on the consideration that the study did not involve any direct interaction with the patients or any impact on their care, ensuring that patient confidentiality and integrity were maintained throughout the study process.

### 2.5. Statistical analysis

The associations between clinical characteristics and pneumonia prognosis were examined through univariate and multivariate logistic regression analyses, with results presented as odds ratios and 95% confidence intervals (CIs). The performance of clinical parameters as predictors of prognosis was assessed using the Receiver Operating Characteristic (ROC) curve analysis. The Area Under the Curve (AUC) was calculated to quantify the predictive accuracy, and the Youden index was employed to identify optimal cutoff points for each parameter. The statistical threshold for significance was set at *P* < .05. All statistical analyses were performed using SPSS Statistics software, version 26 (IBM Corp., Armonk, NY).

## 3. Results

### 3.1. Baseline characteristics of the study population

Table 1 presents the baseline characteristics of the study population, encompassing both clinical and demographic parameters. The study involved a total of 338 children diagnosed with refractory Mycoplasma pneumoniae pneumonia. The distribution of genders was nearly even, with 177 females (52.4%) and 161 males (47.6%). The average age of the participants was 6.07 years, with a standard deviation of 2.7762 years. In terms of educational background of the parents, 168 children (49.7%) had parents with a college degree or higher, while 170 children (50.3%) had parents with a high school education or below. Household income levels were reported as below 10,000 units for 179 children (53%) and above 10,000 units for 159 children (47%).The mean Body Mass Index (BMI) was 16.041 kg/m² with a standard deviation of 2.0367. The study population showed a significant presence of allergic constitution, with 180 children (53.3%) reported as having allergies, and 158 children (46.7%) reported as not having allergies. The duration of fever averaged 5.1755 days, with a standard deviation of 1.0423 days.Laboratory findings revealed a mean WBC count of 6.5099 × 10^9^ cells/L (standard deviation 1.5239), a mean neutrophil count of 4.08 × 10^9^ cells/L (standard deviation 0.98257), and a mean lymphocyte count of 1.9409 × 10^9^ cells/L (standard deviation 0.51149). The mean platelet count was 246.11 × 10^9^ cells/L (standard deviation 51.978). The NLR and PLR were 2.5862 (standard deviation 0.50402) and 123.09 (standard deviation 20.9), respectively. CRP levels averaged 5.4267 mg/L (standard deviation 1.9855), DD averaged 0.41434 mg/L (standard deviation 0.057606), PCT levels averaged 0.21597 ng/mL (standard deviation 0.054258), and LDH levels averaged 200.45 U/L (standard deviation 45.297).

**Table 1:** Baseline characteristics of the study population.

Characteristics	Overall
Gender, n (%)	
Female	177 (52.4%)
Male	161 (47.6%)
Age, mean ± sd	6.0669 ± 2.7762
Parents_Educational_Level, n (%)	
College or above	168 (49.7%)
High school or below	170 (50.3%)
Household_Income, n (%)	
Below 10,000	179 (53%)
Above 10,000	159 (47%)
BMI, mean ± SD	16.041 ± 2.0367
Allergic_Constitution, n (%)	
Yes	180 (53.3%)
No	158 (46.7%)
Duration_of_Fever, mean ± SD	5.1755 ± 1.0423
WBC, mean ± SD	6.5099 ± 1.5239
Neutrophil_Count, mean ± SD	4.08 ± 0.98257
Lymphocyte_Count, mean ± SD	1.9409 ± 0.51149
Platelet_Count, mean ± SD	246.11 ± 51.978
NLR, mean ± SD	2.5862 ± 0.50402
PLR, mean ± SD	123.09 ± 20.9
CRP, mean ± SD	5.4267 ± 1.9855
DD, mean ± SD	0.41434 ± 0.057606
PCT, mean ± SD	0.21597 ± 0.054258
LDH, mean ± SD	200.45 ± 45.297

Gender, Parents’ Educational Level, Household Income, and Allergic Constitution are presented as number and percentage (n [%]). Age, BMI (Body Mass Index), duration of fever, WBC (white blood cell count), neutrophil count, lymphocyte count, platelet count, NLR (neutrophil to lymphocyte ratio), PLR (platelet to lymphocyte ratio), CRP (C-reactive protein), DD (D-Dimer), PCT (procalcitonin), and LDH (lactate dehydrogenase) are expressed as mean ± standard deviation (SD).

Units for variables are—age: years, BMI (Body Mass Index): kg/m², duration of fever: days, WBC (white blood cell count): ×10^9^ cells/L, neutrophil count: ×10^9^ cells/L, lymphocyte count: ×10^9^ cells/L, platelet count: ×10^9^ cells/L, NLR (neutrophil to lymphocyte ratio): ratio, PLR (platelet to lymphocyte ratio): ratio, CRP (C-reactive protein): mg/L, DD (D-Dimer): mg/L, PCT (procalcitonin): ng/mL, LDH (lactate dehydrogenase): U/L. Gender, parents’ educational level, household income, and allergic constitution are presented as number and percentage (n [%]).

BMI = Body Mass Index, CRP = C-reactive protein, DD = D-Dimer, LDH = lactate dehydrogenase, NLR = neutrophil to lymphocyte ratio, PCT = procalcitonin, PLR = platelet to lymphocyte ratio, WBC = white blood cell count.

### 3.2. Comparison of clinical characteristics between patients with good and poor prognosis

Table 2 provides a detailed comparison of demographic and clinical characteristics between 2 groups of patients: those with a good prognosis and those with a poor prognosis. The data include 241 patients in the good prognosis group and 97 in the poor prognosis group. For gender, the good prognosis group comprises 119 females (35.2%) and 122 males (36.1%), while the poor prognosis group includes 58 females (17.2%) and 39 males (11.5%), with a *P*-value of 0.083 suggesting no significant gender-based difference in prognosis. Age is reported as mean ± standard deviation, with the good prognosis group averaging 5.802 ± 2.8293 years and the poor prognosis group 6.7249 ± 2.5359 years; this difference is statistically significant (*P*-value = 0.006). Parental educational levels are categorized into “College or Above” and “High School or Below,” with 123 (36.4%) parents from the good prognosis group and 45 (13.3%) from the poor group having attended college or above, yielding a *P*-value of 0.440. Household income is divided into 2 categories: below and above 10,000 units, with 132 (39.1%) in the lower income bracket in the good prognosis group compared to 47 (13.9%) in the poor prognosis group, and 109 (32.2%) versus 50 (14.8%) in the higher income bracket respectively, with a *P*-value of 0.292. The BMI is expressed as mean ± standard deviation, with the good prognosis group having a BMI of 15.731 ± 1.9732 kg/m², and the poor prognosis group with 16.812 ± 1.9967 kg/m², which is statistically significant (*P*-value < 0.001). For allergic constitution, 121 (35.8%) patients with good prognosis and 59 (17.5%) with poor prognosis report having an allergic constitution, with a *P*-value of 0.077. Clinical indicators such as duration of fever, WBC count, neutrophil count, lymphocyte count, platelet count, NLR, PLR, CRP, DD, PCT, and LDH are also reported. The duration of fever averages 4.9101 ± 0.98282 days in the good prognosis group and 5.8348 ± 0.88469 days in the poor prognosis group, significant at *P*-value < 0.001. Similarly, other laboratory values show significant differences, indicating varying levels of inflammation and other physiological responses between the groups.

**Table 2:** Comparison of clinical characteristics between patients with good and poor prognosis.

Characteristics	Good	Poor	*P*-value
n	241	97	
Gender, n (%)			.083
Female	119 (35.2%)	58 (17.2%)	
Male	122 (36.1%)	39 (11.5%)	
Age, mean ± SD	5.802 ± 2.8293	6.7249 ± 2.5359	.006
Parents_Educational_Level, n (%)			.440
College or above	123 (36.4%)	45 (13.3%)	
High school or below	118 (34.9%)	52 (15.4%)	
Household_Income, n (%)			.292
Below 10,000	132 (39.1%)	47 (13.9%)	
Above 10,000	109 (32.2%)	50 (14.8%)	
BMI, mean ± SD	15.731 ± 1.9732	16.812 ± 1.9967	<.001
Allergic_Constitution, n (%)			.077
Yes	121 (35.8%)	59 (17.5%)	
No	120 (35.5%)	38 (11.2%)	
Duration_of_Fever, mean ± SD	4.9101 ± 0.98282	5.8348 ± 0.88469	<.001
WBC, mean ± SD	6.346 ± 1.5511	6.917 ± 1.3794	.002
Neutrophil_Count, mean ± SD	3.8995 ± 0.96125	4.5284 ± 0.89116	<.001
Lymphocyte_Count, mean ± SD	2.0064 ± 0.49652	1.7781 ± 0.51432	<.001
Platelet_Count, mean ± SD	250.18 ± 50.825	235.99 ± 53.677	.023
NLR, mean ± SD	2.5147 ± 0.48732	2.7639 ± 0.50329	<.001
PLR, mean ± SD	120.16 ± 20.24	130.36 ± 20.837	<.001
CRP, mean ± SD	5.088 ± 1.8666	6.2682 ± 2.031	<.001
DD, mean ± SD	0.3975 ± 0.051404	0.4562 ± 0.050606	<.001
PCT, mean ± SD	0.19919 ± 0.047571	0.25765 ± 0.047063	<.001
LDH, mean ± SD	198.5 ± 43.992	205.3 ± 48.281	.212

The table presents the comparison of demographic and clinical characteristics between patients with a good and poor prognosis. Data are expressed as mean ± standard deviation (SD) for continuous variables and number and percentage (n [%]) for categorical variables. The *P* values indicate the level of statistical significance between the 2 groups.

Units for variables are as follows: age: years, BMI (Body Mass Index): kg/m², duration of fever: days, WBC (white blood cell count): ×10^9^ cells/L, neutrophil count: ×10^9^ cells/L, lymphocyte count: ×10^9^ cells/L, platelet count: ×10^9^ cells/L, NLR (neutrophil to lymphocyte ratio): ratio, PLR (platelet to lymphocyte ratio): ratio, CRP (C-reactive protein): mg/L, DD (D-Dimer): mg/L, PCT (procalcitonin): ng/mL, LDH (lactate dehydrogenase): U/L. Gender, parents’ educational level, household income, and allergic constitution are presented as number and percentage (n [%]).

BMI = Body Mass Index, CRP = C-reactive protein, DD = D-Dimer, LDH = lactate dehydrogenase, NLR = neutrophil to lymphocyte ratio, PCT = procalcitonin, PLR = platelet to lymphocyte ratio, WBC = white blood cell count.

### 3.3. Univariate and multivariate analysis of factors associated with disease prognosis

**Figure 1.**
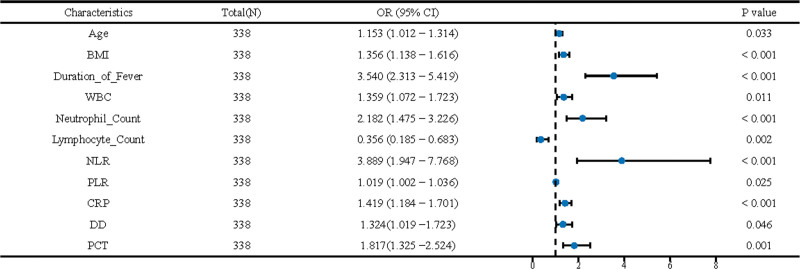
Forest plot depicting the odds ratios (ORs) and 95% confidence intervals (CIs) for various clinical parameters associated with the prognosis of refractory Mycoplasma pneumoniae pneumonia in children. The horizontal lines represent the 95% CIs, the dots indicate the point estimate of the ORs, and the *P* values are provided to show the level of statistical significance. Parameters that have CIs not crossing the vertical line of no effect (OR = 1) are considered statistically significant. The plot illustrates the individual impact of each parameter, with age, BMI, duration of fever, WBC, neutrophil count, lymphocyte count, NLR, PLR, CRP, DD, and PCT included in the multivariate logistic regression model, guiding towards an informed prognosis assessment. BMI = Body Mass Index, CRP = C-reactive protein, DD = D-Dimer, NLR = neutrophil to lymphocyte ratio, PCT = procalcitonin, WBC = white blood cell count.

Table 3 offers a comprehensive analysis elucidating the associations between various demographic and clinical characteristics with the prognosis of the disease under study. The table includes data for 338 patients. For each characteristic, the Odds Ratios (OR) with 95% confidence intervals (CIs) and *P* values are provided, indicating the strength and direction of the associations. In the univariate analysis, gender is represented with males having an OR of 0.656 (95% CI: 0.407–1.058) compared to females, who serve as the reference category. The age of the patients is shown to influence prognosis significantly, with an OR of 1.130 (95% CI: 1.035–1.234) per year increase. Other variables such as parental educational level and household income are examined but do not show significant associations in univariate analysis. BMI shows a significant association, with an OR of 1.317 (95% CI: 1.161–1.493) per unit increase. For allergic constitution, those with allergies have an OR of 1.540 (95% CI: 0.953–2.487) compared to those without. Duration of fever significantly affects prognosis, with an OR of 2.963 (95% CI: 2.166–4.054) per day increase. Biological markers such as WBC, neutrophil count, lymphocyte count, and platelet count are also analyzed. Neutrophils and lymphocytes show strong associations with disease prognosis, with significant odds ratios in both analyses. Additionally, NLR, PLR, CRP, DD, and PCT are significant predictors in both univariate and multivariate analyses. Multivariate analysis adjusts for potential confounders and largely confirms the significance of many factors seen in univariate analysis. For instance, the duration of fever shows an even higher OR of 3.540 (95% CI: 2.313–5.419) in multivariate analysis. Similarly, biological markers such as CRP and PCT remain significant with ORs of 1.419 (95% CI: 1.184–1.701) and 1.817 (95% CI: 1.325–2.524), respectively. Forest plots are shown in Figure 1.

**Table 3:** Univariate and multivariate analysis of factors associated with disease prognosis.

Characteristics	Total(N)	Univariate analysis		Multivariate analysis	
		Odds ratio (95% CI)	*P*-value	Odds ratio (95% CI)	*P*-value
Gender	338				
Female	177	Reference		Reference	
Male	161	0.656 (0.407–1.058)	.084	0.619 (0.314–1.222)	.167
Age	338	1.130 (1.035–1.234)	**.006**	1.153 (1.012–1.314)	**.033**
Parents_Educational_Level	338				
High school or below	170	Reference			
College or above	168	0.830 (0.518–1.331)	.440		
Household_Income	338				
Below 10,000	179	Reference			
Above 10,000	159	1.288 (0.803–2.066)	.293		
BMI	338	1.317 (1.161–1.493)	**<.001**	1.356 (1.138–1.616)	**<.001**
Allergic_Constitution	338				
No	158	Reference		Reference	
Yes	180	1.540 (0.953–2.487)	.078	1.526 (0.776–3.003)	.221
Duration_of_Fever	338	2.963 (2.166–4.054)	**<.001**	3.540 (2.313–5.419)	**<.001**
WBC	338	1.293 (1.098–1.523)	**.002**	1.359 (1.072–1.723)	**.011**
Neutrophil_Count	338	2.065 (1.563–2.728)	**<.001**	2.182 (1.475–3.226)	**<.001**
Lymphocyte_Count	338	0.404 (0.248–0.658)	**<.001**	0.356 (0.185–0.683)	**.002**
Platelet_Count	338	0.995 (0.990–0.999)	**.024**	0.999 (0.992–1.006)	.767
NLR	338	2.821 (1.702–4.677)	**<.001**	3.889 (1.947–7.768)	**<.001**
PLR	338	1.025 (1.012–1.037)	**<.001**	1.019 (1.002–1.036)	**.025**
CRP	338	1.376 (1.206–1.569)	**<.001**	1.419 (1.184–1.701)	**<.001**
DD	338	1.504 (1.132–2.014)	**.023**	1.324 (1.019–1.723)	**.046**
PCT	338	2.012 (1.478–2.817)	**<.001**	1.817 (1.325–2.524)	**.001**
LDH	338	1.003 (0.998–1.009)	.212		

This table presents the results from both univariate and multivariate logistic regression analyses, examining the association of various demographic and clinical characteristics with disease prognosis. Odds ratios (OR) with 95% confidence intervals (CI) are reported to indicate the strength and direction of these associations, alongside their statistical significance (*P* value).

Reference categories are used for categorical variables to compute ORs. For gender, “Female” serves as the reference category. Similarly, for allergic constitution, “No” is considered the reference.

Units for variables are as follows: Age: years, BMI (Body Mass Index): kg/m², duration of fever: days, WBC (white blood cell count): ×10^9^ cells/L, neutrophil count: ×10^9^ cells/L, lymphocyte count: ×10^9^ cells/L, platelet count: ×10^9^ cells/L, NLR (neutrophil to lymphocyte ratio): ratio, PLR (platelet to lymphocyte ratio): ratio, CRP (C-reactive protein): mg/L, DD (D-Dimer): mg/L, PCT (procalcitonin): ng/mL, LDH (lactate dehydrogenase): U/L. Odds ratios (OR) are reported with 95% confidence intervals (CI).

BMI = Body Mass Index, CRP = C-reactive protein, DD = D-Dimer, LDH = lactate dehydrogenase, NLR = neutrophil to lymphocyte ratio, PCT = procalcitonin, PLR = platelet to lymphocyte ratio, WBC = white blood cell count.

The bold values in Table 3 indicate statistically significant results, typically with a *P*-value <0.05. These values highlight key findings that are significantly different or noteworthy in the context of the analysis.

### 3.4. Diagnostic performance of clinical parameters and the predictive model in children with RMPP

**Figure 2.**
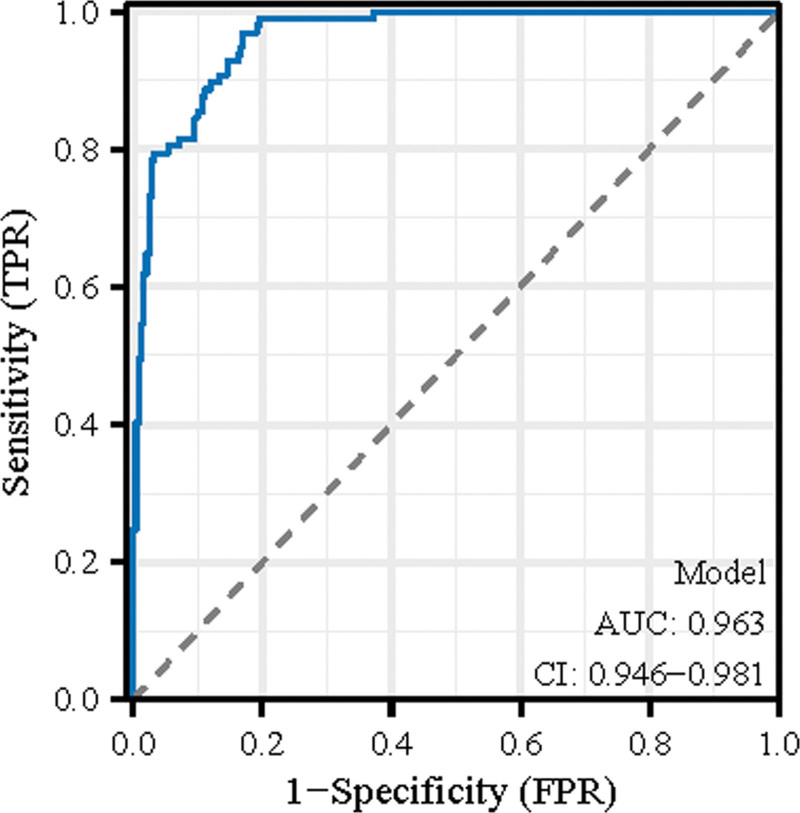
ROC curve illustrating the performance of the predictive model for refractory *Mycoplasma pneumoniae* pneumonia in children. The model shows an AUC (area under the curve) of 0.963, indicating an excellent diagnostic ability. The 95% confidence interval (CI) for the AUC ranges from 0.946 to 0.981, suggesting a high level of precision in the model’s predictive power. The curve approaches the top-left corner of the graph, reflecting a high true positive rate (TPR) with a low false positive rate (FPR), which denotes high sensitivity and specificity of the model. ROC = receiver operating characteristic.

Table 4 assesses the diagnostic performance of various clinical parameters and a predictive model in identifying children with RMPP. The table provides the Area Under the ROC Curve (AUC), 95% CI, optimal cutoff values, and the corresponding specificity and sensitivity percentages for each parameter. Specific clinical parameters listed include age, BMI, duration of fever, WBC count, neutrophil count, lymphocyte count, NLR, PLR, CRP, DD, and PCT. Each parameter’s diagnostic effectiveness is quantified by its AUC, showcasing the ability to distinguish between children with and without refractory pneumonia. For example, the AUC for age is 0.597 with a CI of 0.532 to 0.662, and a cutoff value of 4.019 years, resulting in a specificity of 30.29% and a sensitivity of 88.66%. The parameter showing the highest diagnostic utility is the predictive model itself, with an AUC of 0.963 (CI: 0.946–0.981), a very high specificity of 82.988%, and a sensitivity of 96.907%. This model encapsulates the combined predictive capabilities of the included parameters. The ROC curve plot is shown in Figure 2.

**Table 4:** Diagnostic performance of clinical parameters and the predictive model in children with refractory *Mycoplasma pneumoniae* pneumonia.

Parameters	AUC	95% CI	Cutoff value	Specificity (%)	Sensitivity (%)
Age	0.597	0.532–0.662	4.019	0.3029	0.8866
BMI	0.651	0.587–0.715	16.587	0.6971	0.53608
Duration_of_Fever	0.757	0.701–0.812	5.4225	0.70124	0.71134
WBC	0.597	0.532–0.661	5.3725	0.29046	0.89691
Neutrophil_Count	0.687	0.626–0.747	4.2015	0.64315	0.69072
Lymphocyte_Count	0.629	0.562–0.696	1.9795	0.56432	0.69072
NLR	0.653	0.590–0.717	2.733	0.6888	0.56701
PLR	0.642	0.576–0.708	132.94	0.76349	0.50515
CRP	0.663	0.599–0.728	5.1685	0.52697	0.73196
DD	0.793	0.742–0.845	0.4195	0.65975	0.80412
PCT	0.805	0.756–0.855	0.2165	0.63485	0.81443
Model	0.963	0.946–0.981	-	0.82988	0.96907

This table evaluates the diagnostic performance of various clinical parameters and a predictive model for identifying children with refractory *Mycoplasma pneumoniae* pneumonia. The diagnostic accuracy is assessed using the area under the receiver operating characteristic (ROC) curve (AUC), with corresponding 95% confidence intervals (CI). Additionally, optimal cutoff values are determined alongside their specificity and sensitivity percentages.

Units for variables are as follows: Age: years, BMI (Body Mass Index): kg/m², duration of fever: days, WBC (white blood cell count): ×10^9^ cells/L, neutrophil count: ×10^9^ cells/L, lymphocyte count: ×10^9^ cells/L, platelet count: ×10^9^ cells/L, NLR (neutrophil to lymphocyte ratio): ratio, PLR (platelet to lymphocyte ratio): ratio, CRP (C-reactive protein): mg/L, DD (D-Dimer): mg/L, PCT (procalcitonin): ng/mL.

The “Model” row represents the combined predictive performance of the identified parameters within the constructed predictive model for refractory *Mycoplasma pneumoniae* pneumonia in children.

Cutoff values are provided for each parameter, offering a threshold that optimizes the balance between specificity (true negative rate) and sensitivity (true positive rate) for the diagnosis of refractory *Mycoplasma pneumoniae* pneumonia.

AUC = area under the curve, BMI = Body Mass Index, CI = confidence interval, CRP = C-reactive protein, DD = D-Dimer, NLR = neutrophil to lymphocyte ratio, PCT = procalcitonin, PLR = platelet to lymphocyte ratio, WBC = white blood cell count.

## 4. Discussion

Our study contributes to the burgeoning field of predictive modeling for RMPP in children, a clinical challenge that has garnered increasing attention due to its complexity and the significant morbidity it can cause. By integrating clinical, laboratory, and radiological data to develop a comprehensive predictive model, our findings resonate with and build upon the pioneering work of Bi et al (2021), Cheng et al (2021), Zhang et al (2023), and Cheng et al (2020), each of whom has contributed valuable tools and insights for early prediction and management of RMPP and its complications.

Bi et al (2021) developed an early predictive scale for RMPP, identifying 6 prognostic indicators that closely align with our findings. Their scale, validated in both retrospective and prospective cohorts, demonstrates the feasibility and utility of early prediction in standard clinical practice.^[21]^ Our model expands on this foundation by incorporating a broader array of variables, enhancing its predictive capacity and clinical applicability.

Similarly, the nomogram models developed by Cheng et al (2021) for predicting the risk of bronchitis obliterans^[22]^ and by Zhang et al (2023) for plastic bronchitis underscore the significance of early,^[23]^ accurate risk stratification in RMPP. These studies highlight specific clinical and laboratory markers, including WBC count and LDH levels, which emerged as significant predictors in our analysis as well. This convergence of findings underscores the critical role of inflammatory and immune response markers in the pathophysiology of RMPP and its severe complications.

Furthermore, the study by Cheng et al (2020) reinforces the importance of simplicity and usability in predictive tools for RMPP. Their nomogram, which also identified LDH and neutrophil ratio as significant predictors, exemplifies the potential of such models to facilitate early recognition and intervention in RMPP.^[24]^ Our model mirrors this approach, emphasizing ease of use and clinical relevance to aid physicians in the timely management of at-risk children.

The collective insights from these studies, together with our own, highlight several key themes in the early prediction and management of RMPP. First, the consistent identification of specific predictors across different models attests to their fundamental role in the disease process and their potential utility in clinical practice. Second, the development and validation of these models underscore the feasibility of integrating predictive modeling into the routine care of children at risk for RMPP, potentially transforming the approach to this challenging clinical scenario.

In synthesizing these contributions, our study not only validates the significance of previously identified predictors but also introduces additional variables into the predictive framework, offering a more nuanced and comprehensive tool for clinicians. By doing so, we aim to enhance the precision of RMPP prognosis and, ultimately, to facilitate more targeted and effective interventions to mitigate the impact of this disease on affected children.

Additionally, our study acknowledges recent advancements in the identification of biomarkers for *M pneumoniae* infections. Notably, the research conducted by Papan et al (2023) introduces a combinatorial host-response biomarker signature (BV score) and its subanalytes TRAIL, IP-10, and CRP, which differentiate bacterial from viral etiologies in children with community-acquired pneumonia.^[25]^ This study demonstrates the potential utility of these biomarkers in enhancing diagnostic precision. The BV score, in particular, provides a nuanced approach to understanding the host response, which is crucial in *M pneumoniae* due to its unique interplay between bacterial and viral infection characteristics.

In light of these findings, incorporating such biomarkers into future iterations of our predictive model could refine our ability to stratify risk and tailor interventions more effectively for RMPP. These newer markers, as detailed by Papan et al, may offer additional layers of diagnostic clarity and could be instrumental in addressing some of the limitations noted in our current approach, such as the need for external validation and the potential influence of unrecognized pathophysiological variations.

Our study, while offering valuable insights into predicting RMPP in children, is subject to several limitations that warrant consideration. Firstly, its retrospective nature may introduce selection bias, as the data were collected from past records, which could affect the generalizability of our findings. Secondly, being conducted at a single center, the study’s results might not be directly applicable to other settings with different patient demographics or treatment protocols, thus limiting the external validity of our predictive model. Additionally, our study did not account for macrolide resistance directly, a significant factor in RMPP outcomes, which could influence the applicability of the predictive model in varying clinical contexts where antibiotic resistance patterns differ. Lastly, the model’s reliance on certain laboratory and clinical parameters might not capture the full spectrum of factors influencing RMPP severity, such as genetic predispositions or environmental influences, suggesting the need for further research incorporating a broader range of predictors. Future studies, ideally multicenter and prospective in design, are necessary to validate and refine our predictive model, enhance its accuracy, and confirm its utility across diverse clinical settings.

In conclusion, our study complements and extends the existing literature on predictive modeling for RMPP in the pediatric population. By integrating a diverse array of clinical and laboratory markers, we have developed a comprehensive model that holds promise for the early identification of children at risk for severe outcomes. This model not only reflects the complexity of RMPP but also offers a pragmatic tool for clinicians to tailor interventions more effectively. Moving forward, the validation of our findings across broader cohorts and the integration of predictive models into clinical practice will be crucial steps toward mitigating the impact of this challenging condition on affected children.

## Acknowledgments

We would like to express our sincere gratitude to all individuals who contributed to the completion of this study.

## Author contributions

**Conceptualization:** Hong Pei, Hongli Luo.

**Data curation:** Hong Pei, Hongli Luo.

**Formal analysis:** Hong Pei.

**Investigation:** Hong Pei.

**Methodology:** Hong Pei.

**Software:** Hongli Luo.

**Supervision:** Hongli Luo.

**Writing – original draft:** Hongli Luo.

**Writing – review & editing:** Hongli Luo.

## References

[1] Medjo B Atanaskovic-Markovic M Radic S Nikolic D Lukac M Djukic S. Mycoplasma pneumoniae as a causative agent of community-acquired pneumonia in children: clinical features and laboratory diagnosis. Ital J Pediatr. 2014;40:104. 25518734 10.1186/s13052-014-0104-4 PMC4279889

[2] Aguilera-Alonso D López Ruiz R Centeno Rubiano J. Epidemiological and clinical analysis of community-acquired *Mycoplasma* pneumonia in children from a Spanish population, 2010-2015. An Pediatr (Engl Ed). 2019;91:21–9. 10.1016/j.anpede.2019.01.003 PMC7146767 32289046

[3] Meyer Sauteur PM Theiler M Buettcher M Seiler M Weibel L Berger C. Frequency and clinical presentation of mucocutaneous disease due to mycoplasma pneumoniae infection in children with community-acquired pneumonia. JAMA Dermatol. 2020;156:144–50. 31851288 10.1001/jamadermatol.2019.3602 PMC6990853

[4] Zhou Y Wang J Chen W. Impact of viral coinfection and macrolide-resistant mycoplasma infection in children with refractory *Mycoplasma pneumoniae* pneumonia. BMC Infect Dis. 2020;20:633. 32847534 10.1186/s12879-020-05356-1 PMC7447613

[5] Xie Q Zhang X Cui W Pang Y. Construction of a nomogram for identifying refractory *Mycoplasma pneumoniae* pneumonia among macrolide-unresponsive *Mycoplasma pneumoniae* pneumonia in children. J Inflamm Res. 2022;15:6495–504. 36474517 10.2147/JIR.S387809 PMC9719700

[6] Song Z Jia G Luo G Han C Zhang B Wang X. Global research trends of *Mycoplasma pneumoniae* pneumonia in children: a bibliometric analysis. Front Pediatr. 2023;11:1306234. 38078315 10.3389/fped.2023.1306234 PMC10704248

[7] Tong L Huang S Zheng C Zhang Y Chen Z. Refractory *Mycoplasma pneumoniae* pneumonia in children: early recognition and management. J Clin Med. 2022;11:2824. 35628949 10.3390/jcm11102824 PMC9144103

[8] Liu C Wang R Ge S Wang B Li S Yan B. Research status and challenges of *Mycoplasma pneumoniae* pneumonia in children: a bibliometric and visualization analysis from 2011 to 2023. Medicine (Baltimore). 2024;103:e37521. 38489686 10.1097/MD.0000000000037521 PMC10939570

[9] Shen F Dong C Zhang T. Development of a nomogram for predicting refractory *Mycoplasma pneumoniae* pneumonia in children. Front Pediatr. 2022;10:813614. 35281240 10.3389/fped.2022.813614 PMC8916609

[10] Chen J Xi Z Shi Y. Highly homogeneous microbial communities dominated by *Mycoplasma pneumoniae* instead of increased resistance to macrolide antibiotics is the characteristic of lower respiratory tract microbiome of children with refractory *Mycoplasma pneumoniae* pneumonia. Transl Pediatr. 2021;10:604–15. 33850819 10.21037/tp-20-404 PMC8039789

[11] Gan T Yu J He J. miRNA, lncRNA and circRNA: targeted molecules with therapeutic promises in *Mycoplasma pneumoniae* infection. Arch Microbiol. 2023;205:293. 37477725 10.1007/s00203-023-03636-3

[12] Fan F Lv J Yang Q Jiang F. Clinical characteristics and serum inflammatory markers of community-acquired mycoplasma pneumonia in children. Clin Respir J. 2023;17:607–17. 37142438 10.1111/crj.13620 PMC10363789

[13] Klings ES Steinberg MH. Acute chest syndrome of sickle cell disease: genetics, risk factors, prognosis, and management. Expert Rev Hematol. 2022;15:117–25. 35143368 10.1080/17474086.2022.2041410

[14] Sun JH Sun F Yan B Li JY Xin L. Data mining and systematic pharmacology to reveal the mechanisms of traditional Chinese medicine in *Mycoplasma pneumoniae* pneumonia treatment. Biomed Pharmacother. 2020;125:109900. 32028237 10.1016/j.biopha.2020.109900

[15] Li J Luu LDW Wang X. Metabolomic analysis reveals potential biomarkers and the underlying pathogenesis involved in *Mycoplasma pneumoniae* pneumonia. Emerg Microbes Infect. 2022;11:593–605. 35094669 10.1080/22221751.2022.2036582 PMC8865114

[16] Jang MS Kim BG Kim J. Prediction model for prolonged fever in patients with *Mycoplasma pneumoniae* pneumonia: a retrospective study of 716 pediatric patients. BMC Pulm Med. 2021;21:168. 34006256 10.1186/s12890-021-01534-2 PMC8130327

[17] Li M Wei X Zhang SS. Recognition of refractory Mycoplasma pneumoniae pneumonia among *Myocoplasma pneumoniae* pneumonia in hospitalized children: development and validation of a predictive nomogram model. BMC Pulm Med. 2023;23:383. 37817172 10.1186/s12890-023-02684-1 PMC10566172

[18] Chen Q Hu T Wu L Chen L. Clinical features and biomarkers for early prediction of refractory *Mycoplasma pneumoniae* pneumonia in children. Emerg Med Int. 2024;2024:9328177. 38222094 10.1155/2024/9328177 PMC10787049

[19] Gong H Sun B Chen Y Chen H. The risk factors of children acquiring refractory *Mycoplasma pneumoniae* pneumonia: a meta-analysis. Medicine (Baltimore). 2021;100:e24894. 33725960 10.1097/MD.0000000000024894 PMC7982158

[20] Shao SL Cong HY Wang MY Liu P. The diagnostic roles of neutrophil in bloodstream infections. Immunobiology. 2020;225:151858. 31836303 10.1016/j.imbio.2019.10.007

[21] Bi Y Zhu Y Ma X. Development of a scale for early prediction of refractory *Mycoplasma pneumoniae* pneumonia in hospitalized children. Sci Rep. 2021;11:6595. 33758243 10.1038/s41598-021-86086-5 PMC7987979

[22] Cheng Q Zhang H Shang Y. Clinical features and risk factors analysis of bronchitis obliterans due to refractory *Mycoplasma pneumoniae* pneumonia in children: a nomogram prediction model. BMC Infect Dis. 2021;21:1085. 34674642 10.1186/s12879-021-06783-4 PMC8529771

[23] Zhang H Yang J Zhao W. Clinical features and risk factors of plastic bronchitis caused by refractory *Mycoplasma pneumoniae* pneumonia in children: a practical nomogram prediction model. Eur J Pediatr. 2023;182:1239–49. 36633659 10.1007/s00431-022-04761-9 PMC10023623

[24] Cheng S Lin J Zheng X. Development and validation of a simple-to-use nomogram for predicting refractory *Mycoplasma pneumoniae* pneumonia in children. Pediatr Pulmonol. 2020;55:968–74. 32040888 10.1002/ppul.24684

[25] Papan C Sidorov S Greiter B. Combinatorial host-response biomarker signature (BV score) and its subanalytes TRAIL, IP-10, and CRP in children with *Mycoplasma pneumoniae* community-acquired pneumonia. J Infect Dis. 2023;229:jiad573. Advance online publication. 10.1093/infdis/jiad573 PMC11326813 38092364

